# Convalescent plasma treatment for patients of 80 years and older with COVID-19 pneumonia

**DOI:** 10.1186/s12877-021-02447-9

**Published:** 2021-10-18

**Authors:** Iñigo Romon, Juan J. Dominguez-Garcia, Jose L. Arroyo, Borja Suberviola, Itxasne Cabezón, Beatriz Abascal, Cristina Baldeón, Amalia Cuesta, Raquel Portilla, Elena Casuso, Enrique Ocio, Montserrat Briz

**Affiliations:** 1 Hematology and Hemotherapy Service, Hospital Universitario Marqués de Valdecilla, Avenida Valdecilla, s/n, 39008 Santander, Spain; 2 Banco de Sangre y Tejidos de Cantabria, 39121 Liencres, Spain; 3 Intensive Care Service, Hospital Universitario Marqués de Valdecilla, 39008 Santander, Spain; 4 Infectious Diseases Service, Hospital Universitario Marqués de Valdecilla, 39008 Santander, Spain; 5 Pneumology Service, Hospital Universitario Marqués de Valdecilla, 39008 Santander, Spain; 6 Internal Medicine Service, Hospital Universitario Marqués de Valdecilla, 39008 Santander, Spain; 7 Haematology Service, Hospital de Sierrallana, 39300 Torrelavega, Spain; 8 Internal Medicine Service, Hospital de Sierrallana, 39300 Torrelavega, Spain; 9 Internal Medicine Service, Hospital de Laredo, 39770 Laredo, Spain

**Keywords:** COVID-19, Convalescent plasma, Older adults, Transfusion

## Abstract

**Background:**

Older patients, frequently with multiple comorbidities, have a high mortality from COVID-19 infection. Convalescent plasma (CP) is a therapeutic option for these patients. Our objective is to retrospectively evaluate the efficacy and adverse events of CP treatment in this population group.

**Methods:**

Forty one patients over 80 years old with COVID-19 pneumonia received CP added to standard treatment, 51.2% with high anti-SARS-CoV-2 IgG titers and 48.8% with low titers. Median time between the onset of symptoms and the infusion of plasma was 7 days (IQR 4–10). A similar group of 82 patients who received only standard treatment, during a period in which CP was not available, were selected as a control group.

**Results:**

In-hospital mortality was 26.8% for controls and 14.6% for CP patients (*P* = 0.131) and ICU admission was 8.5% for controls and 4.9% for CP patients (*P* = 0.467). Mortality tended to be lower in the high-titer group (9.5%) than in the low-titer group (20%), and in patients transfused within the first 7 days of symptom onset (10%) than in patients transfused later (19.1%), although the differences were not statistically significant (*P* = 0.307 and *P* = 0.355 respectively). There was no difference in the length of hospitalization. No significant adverse events were associated with CP treatment.

**Conclusions:**

Convalescent plasma treatment in patients over 80 years old with COVID-19 pneumonia was well tolerated but did not present a statistically significant difference in hospital mortality, ICU admission, or length of hospitalization. The results should be interpreted with caution as only half the patients received high-titer CP and the small number of patients included in the study limits the statistical power to detect significant differences.

**Trial registration:**

CEIm Cantabria # 2020.127.

## Background

The COVID-19 pandemic has created an unprecedented clinical situation in which patients over 65 years of age are more severely affected [1–3]. Chronic conditions, such as cardiovascular diseases, hypertension, obesity, cognitive impairment or diabetes are major risk factors for mortality in patients with COVID-19 [1, 3, 4]. Residing in long term care facilities (LTCF), where older people with multiple comorbidities live in close contact, facilitating virus transmission, can also be a risk [5].

Older patients, over 80 years of age, often residing in LTCF and affected by multiple comorbidities, have suffered the highest mortality rate, ranging from 15 to over 50% [1–3, 5–7]. In fact, the country-specific case fatality rate is predominantly determined by the proportion of older individuals affected [8]. Accordingly, in Europe, where life expectancy usually exceeds 80 years, COVID-19 infection has had a strong impact with high mortality.

In addition, older patients are frequently not candidates for admission to Intensive Care Units (ICU), due to their multiple comorbidities and their high mortality rate, reaching 70–80% [6, 9].

COVID-19 convalescent plasma (CP) is a source of antiviral neutralizing antibodies [10] and has been used to treat hospitalized patients.

The US Food and Drug Administration (FDA), given the lack of effective treatments, granted CP Emergency Use Authorization. Conditions have recently been revised and an early administration of high-titer CP is currently required [11]. This strategy was also implemented by the European Union [12].

In 2020, a CP program was started for the treatment of COVID-19 patients with pneumonia in our region. In this paper we report the results of an interim analysis of the program’s development, focusing on patients over 80 years old, the population group with the highest COVID-19 induced mortality in the region.

## Methods

This study analyses the effect of CP administration to hospitalized adults over 80 years old with pneumonia and compares the results against a control group receiving standard treatment.

### Convalescent plasma treatment protocol

In 2020, a CP prospective study program promoted by the Regional Health Service was started for the treatment of COVID-19 patients with pneumonia recruited at the three regional hospitals (one academic and two community hospitals). Authorization from the Medical Ethics Committee was obtained.

The use of CP had previously been authorized by the National Council for Transfusion Safety [13] and the European Commission [12]. The use of CP was authorized under the provision that a strict vigilance of adverse events and of clinical results was to be maintained. Hospitals and Blood collection centers could manage their own donor base of convalescent patients, and guarantee that donations had COVID-19 antibodies, giving always donations with the highest titers available. A serum sample of all donations had to be kept for future analysis. To ensure that, all patients had to be prospectively registered on an anonymized database managed by the Spanish Government, that would in turn transfer it to the European Commission. Data regarding the patients’ demographic data, most relevant comorbidities, identification of transfused units and follow up data up to 30 days after transfusion were collected.

Adult patients were treated at dedicated COVID-19 wards, irrespective of their age. CP treatment was optional for all adult COVID-19 patients with radiologically confirmed pneumonia, according to the criteria of the patient’s physician, and was compatible with the administration of standard treatment. Informed consent was obtained from the patient or their legal representative. Patients in the ICU were only given plasma during their first day at the ICU. Exclusion criteria were IgA deficiency, known severe adverse reactions to plasma transfusion, and refusal to consent.

The standard of care consisted of steroids (after 7 days of symptoms) and prophylactic heparin. Remdesivir was administered within the first 7 days of symptom onset, and tocilizumab was administered with raised IL-6 levels. Oxygen, antibiotics and other medical treatments were given as required. Criteria for ICU admittance were the patient’s performance status and a life expectancy of at least 6 months before COVID, not their age as such.

CP treatment consisted of one 300 mL ABO compatible plasma unit administered to each patient. Special attention was paid to the avoidance of fluid overload.

To ensure a uniform implementation of the protocol, the treatment protocol was approved by the Heads of Departments of Infectious Diseases, Internal Medicine, Pneumology and Intensive Care of the hospitals treating COVID patients. Periodical follow up meetings between the treating physicians and the steering hematological team were held.

### Convalescent plasma donations

Plasma donors had to be asymptomatic for at least 14 days after COVID-19 infection prior to donation. Prospective donors complied with the legal donor selection criteria for blood donation.

To prevent transfusion-associated acute lung injury (TRALI), which could worsen or mimic COVID-19 lung damage, plasma donors with previous pregnancies or transfusions, at risk of developing anti-HLA antibodies known to cause TRALI, were screened and excluded when positive.

Donations were initially tested by qualitative enzyme-linked immunosorbent assay (ELISA) for anti-SARS-CoV-2 antibodies. Subsequently, all donations were prospectively tested using the VITROS anti SARS-CoV2 IgG antibody test (Ortho-Clinical Diagnostics), and stored serum samples were retrospectively retested. CP units were classified as high-titer and low-titer according to FDA recommendations [11]. As a result, some donations used during the first weeks of the study were tested retrospectively, this revealed that some patients treated during the first weeks of the study randomly received CP units with lower titers. In a small group of patients, lower-titer CP was given by order of the treating physicians when no other option was available. This possibility had been contemplated by the Directive issued by the National Council for Transfusion Safety [13].

### Patients and controls

For an interim analysis of the study, we analyzed the subgroup of 41 patients over 80 years old, treated with CP between August and December 2020, chosen consecutively from those admitted to hospital during that period. As a control group, we chose a group of 82 patients within the same age range, admitted to hospital during a 6-week period between September and November 2020, when CP was unavailable due to a breach in stock. Patients who died within the first 3 days after admission were excluded as controls. We did not extend the analysis beyond January because of the predominance of the British COVID-19 variant, which was considered a different clinical setting.

All patients had a positive RT-PCR test for SARS-CoV-2 in a nasopharyngeal sample and a compatible chest radiography assessed by a trained radiologist.

Data extracted from the patients’ records included age, sex, residence (home vs. LTCF), comorbidities and arterial oxygen saturation, SaO2/FiO2 (SAFI) and PaO2/FiO2 (PAFI that had been recorded upon hospital admission. Before transfusion, patients in the CP group were assessed via the WHO scale for the improvement of COVID-19, [14] and assessment in the control group took place at the time corresponding to the median number of days between admission and plasma infusion in the CP group. Pharmacological treatment for COVID-19 pneumonia was also recorded.

### Outcomes

The primary outcome was the impact of CP administration on all cause in-hospital mortality rate. Secondary outcomes were the need for ICU admission and length of hospital stay. Adverse events within 24 h of transfusion were also recorded.

### Statistical analysis

Data were recorded on a Microsoft Excel 2008 database (Redmond, Washington, USA) and analysed using the StataIC-16 (College Station, Texas, USA) for the descriptive and the statistical inference, and SPSS Statistics 25 (Brussel, Belgium) application for the survival analysis. Normality, asymmetry and skewness were analyzed with the Shapiro-Wilk normality test and the normal standardized probability plot. Quantitative variables were analyzed by comparing their means with the t-test. Categorical variables were analyzed with Pearson’s chi-squared if samples had a relative frequency higher than five or the Fisher’s exact test if their relative frequency was lower. Survival analysis was performed using the Kaplan-Meier method. Statistical significance was achieved if *p*-value ≤0.05.

For a multivariate analysis, we performed a Cox proportional hazards model analysis to investigate the influence of convalescent plasma treatment on mortality.

## Results

A total of 123 patients over 80 years of age were analyzed (mean age 86.2, SD 4.6), 41 in the CP group and 82 in the control group. Women accounted for 41% of patients (CP 53.7% vs. controls 35.4%) Patients living in LTCF accounted for 20.3%, and the most prevalent comorbidities were hypertension (78.1%), cardiovascular disease (51.2%), dyslipidemia (48.0%), cognitive impairment (31.7%) and diabetes (30.9%), with no differences between groups (Table 1).

**Table 1:** Demographic characteristics, comorbidities, clinical data, treatment and outcomes of the overall sample and each cohort

	Overall Cohort (*n* = 123)	Convalescent Plasma (*n* = 41)	Control Group (*n* = 82)	*p*-value
Age, mean (SD)	86.2 (4.60)	86.7 (5.02)	85.9 (4.39)	0.416
Female sex, n (%)	51 (41.5)	22 (53.7)	29 (35.4)	0.052
Comorbidity				
Mental impairment, n (%)	39 (31.7)	14 (34.2)	25 (30.5)	0.681
Hypertension, n (%)	96 (78.1)	32 (78.1)	64 (78.1)	1.000
Diabetes mellitus, n (%)	38 (30.9)	9 (22.0)	29 (35.4)	0.129
Dyslipidemia, n (%)	59 (48.0)	17 (41.5)	42 (51.2)	0.307
Obesity, n (%)	19 (15.5)	3 (7.3)	16 (19.5)	0.078
Cardiovascular disease, n (%)	63 (51.2)	18 (43.9)	45 (54.9)	0.251
CPD, n (%)	28 (22.8)	8 (19.5)	20 (24.4)	0.543
Current or past smoker, n (%)	24 (19.5)	8 (19.5)	16 (19.5)	1.000
Chronic kidney disease, n (%)	25 (20.3)	7 (17.1)	18 (22.0)	0.526
Cancer, n (%)	34 (27.6)	13 (31.7)	21 (25.6)	0.476
LTCF residents, n (%)	25 (20.3)	8 (19.5)	17 (20.7)	0.874
Days from symptom onset to admission (*)	5 (2–7)	5 (2–7)	5 (3–7)	0.877
Days from symptom onset to CP infusion (*)	N/A	7 (4–10)	N/A	–
Days from admission to CP infusion (*)	N/A	1 (0–2)	N/A	–
SaO_2_ (mmHg) at admission (+)	91.8 (0.42)	92.1 (0.60)	91.7 (0.56)	0.606
Estimated PaO_2_/FiO_2_ at admission (+)	321.5 (5.70)	319.9 (8.83)	322.3 (7.35)	0.848
WHO scale (+)	3.8 (0.06)	3.8 (0.09)	3.8 (0.07)	1.000
Treatment, n (%)				
Anticoagulants	122 (99.2)	41 (100)	81 (98.8)	0.667
Antibiotics	119 (96.8)	39 (95.1)	80 (97.6)	0.472
Glucocorticoids	100 (81.3)	32 (78.1)	68 (82.9)	0.513
Remdesivir	18 (14.6)	13 (31.7)	5 (6.1)	0.000
Tocilizumab	14 (11.4)	7 (17.1)	7 (8.5)	0.160
Anakinra	2 (1.6)	1 (2.4)	1 (1.2)	0.557
Lopinavir/ritonavir	1 (0.8)	1 (2.4)	0 (0.0)	0.333
Outcomes				
In-hospital mortality, n (%)	28 (22.7)	6 (14.6)	22 (26.8)	0.131
ICU admission, n (%)	9 (7.3)	2 (4.9)	7 (8.5)	0.467
LoS, discharged, median (IQR)	11 (9–16)	11 (9–16)	11 (7.5–16)	0.073
LoS, dead, median (IQR)	9 (6–12)	11 (7–22)	8.5 (5–12)	0.067

*SD* Standard Deviation, *CPD* Chronic Pulmonary Disease, *LTCF* Long Term Care Facilities, *SaO2* Arterial oxygen saturation, *FiO2* Fraction of inspired Oxygen, *N/A* Not Applicable, *ICU* Intensive Care Unit, *LoS* Length of Stay, *IQR* Interquartile Range

(*) Expressed as median (Interquartile Range). (+) Expressed as mean (Standard Deviation)

Median time from the onset of symptoms to hospital admission (5 days, IQR 2–7), arterial oxygen saturation, SAFI and PAFI at admission, and WHO scale for COVID pneumonia assessment were comparable between both groups (Table 1).

Most patients in both arms received anticoagulants, antibiotics, and glucocorticoids, and less frequently Tocilizumab, Anakinra or Lopinavir/Ritonavir, with no differences between groups. However, Remdesivir was administered more often to patients in the plasma group (CP 31.7%, vs. controls 6.1%; *P* < 0.001).

Median time from the onset of symptoms and hospital admission to CP administration was 7 (IQR 4–10) and 1 (IQR 0–2) days, respectively. High-titer units were given to 21 patients (51.2%) and low-titer CP to 20 cases (48.8%).

Two mild transfusion adverse events (4.9%) were reported, one patient complained of headache and another had a fever after transfusion, both responding to symptomatic treatment.

### Clinical outcome

The all-cause in-hospital mortality (IHM) rate was 22.8% (28 patients) while 77.2% (95 patients) were discharged. Nine patients (7.3%) were admitted to ICU for mechanical ventilation, five of which died (55.6%). Patients who were not considered candidates for ICU admission received appropriate palliative care where necessary.

The length of stay for discharged patients was 11 days (IQR 9–16), with no differences between CP and control cohorts.

Although the CP group presented a lower IHM rate (CP 14.6% vs. controls 26.8%) and lower ICU admission (CP 4.9% vs. control 8.5%), these differences were not statistically significant (*P* = 0.131 and *P* = 0.467, Overall survival: chi-squared log-Rank: 3.79, *P* = 0.052) (Table 1).

**Fig. 1:**
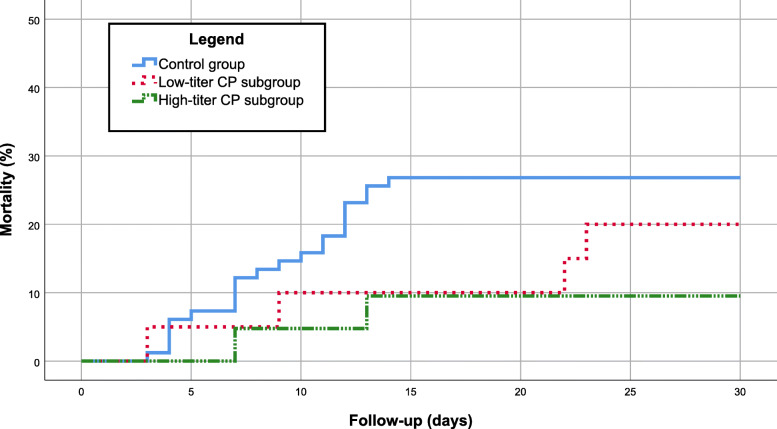
Mortality rate for high and low titer CP subgroups and for the control group

There was a lower mortality trend for the subgroup of 21 patients who received high-titer CP (9.5%) and for the 20 patients who received CP within 6 days of symptom onset (10%), but these differences were not significant (log-Rank: 4.23, *P* = 0.121 and *P* = 0.355 respectively) (Table 2 and Fig. 1).

**Table 2:** In-hospital mortality related to groups, CP titers and days from symptom onset

	Sample Size, n	Mortality, n (%)	*p*-value
Control group	82	22 (26.8)	0.128
Convalescent Plasma group	41	6 (14.6)	
Low-titer CP	20	4 (20.0)	0.307
High-titer CP	21	2 (9.5)	
Symptom onset to CP < 7 days	20	2 (10.0)	0.355
Symptom onset to CP ≥ 7 days	21	4 (19.1)	

*CP* Convalescent Plasma

In a multivariate model using the Cox proportional hazards model, the HR for the influence of CP, adjusted by age, WHO initial status, sex and ICU admittance was 0.887 (*p* = 0.560).

Acute respiratory distress syndrome and subsequent multi-organ failure was the most common cause of death (92.9%). One patient from each group died of retroperitoneal hemorrhage.

Only one patient in the control group was diagnosed with thromboembolic disease (already present at hospital admission).

## Discussion

We describe the results of CP as adjuvant to standard treatment for COVID-19 infection with pneumonia in a group of 41 patients over 80 years of age with multiple comorbidities, and compare them with a control group of 82 similar patients.

Most deaths in both groups were directly attributed to COVID-19 infection. Although patients treated with CP had lower overall in-hospital mortality than controls (14.6% vs. 26.8%) and lower ICU admissions (4.9% vs. 8.5%) the difference was not statistically significant. Similar to other reports [6, 9], the mortality of patients requiring ICU care was very high (55.6%).

The results of studies on CP treatment present apparently discrepant conclusions.

In two randomized studies, the administration of CP in hospitalized patients with pneumonia did not show a better clinical evolution or decrease in mortality [15, 16]. In contrast, a recent randomized study shows that early administration of CP (within 72 h after the onset of symptoms) with high anti-SARS-CoV-2 levels, significantly reduces the progression to severe respiratory disease [17]. Also, a retrospective study of 3082 cases showed that mortality was lower in transfused patients with high titers of anti-SARS-CoV-2 IgG antibodies than in those receiving low-titer CP, with no benefit for patients on mechanical ventilation at the time of transfusion [18]. Similarly, another report describes a significant decrease in mortality in patients receiving high-titer CP within the first 72 h after admission [19]. Results were recently updated establishing that the optimal approach to reduce mortality appears to be the transfusion of high-titer CP within the first 44 h after hospitalization [20].

Discrepancies may be the result of the heterogeneity of the patients selected (time of evolution and severity of the disease) and in the antibody levels of the CP administered.

Our results suggest that CP treatment in this population group does not improve clinical results. However, the absence of statistically significant differences in favor of CP may be due to several factors. First, lack of statistical power because of the small number of patients treated. Second, the late administration of convalescent plasma (median 7 days after the onset of symptoms), as the subgroup of patients receiving CP treatment within 7 days of symptoms had a mortality of 10%, compared with 19% for those who received it later, and 26% for controls. Third, only half of the patients received high-titer CP with a 9.5% mortality, compared with 20% for patients receiving low-titer CP and 26.8% for controls. Finally, we cannot rule out that CP treatment could be ineffective.

A recent report explored the use of CP in 22 older patients living in LTCF, and the overall mortality compared favorably with the Health authorities-reported mortality in LTCF in the same region and period of time [21]. The researchers admit that, given the emergency situation, some of their patients died in their LTCF without receiving adequate treatment or supportive care in a hospital setting. Therefore, it is likely that the control group, even if it had similar baseline characteristics, did not receive the same treatment in the different LTCFs, which may explain the better results in the group treated with CP. In our case, both patients and controls were treated at the same hospitals, by the same professional teams, with homogeneous criteria. In our region there have been no restrictions for patients’ access to hospital or ICU. Some of Franchini’s patients received two or three plasma units, while ours received only one. Moreover, Franchini’s patients had lower obesity, cardiovascular and chronic kidney disease rates than ours.

CP administration was well tolerated, with mild adverse events in 4.9% of the patients, in agreement with a large series in which the incidence of serious transfusion reactions was under 1% [22]. The incidence of thrombotic events was very low, probably because most patients received anticoagulant drugs, usually in prophylactic doses.

Although our study could not demonstrate the effectivity of CP treatment, CP can still play an important role in the treatment of selected patients with impaired immune response [23]. Considering the low incidence of CP adverse events and the scarce therapeutic alternatives for this group of older, high-risk patients, it is worth conducting further studies. To optimize treatment, CP must contain high anti-SARS-CoV-2 titers and should be administered early in the course of the disease.

Our study has some limitations. 1) Being a retrospective study, some information may have been lost if it was not reflected in medical records, such as the existence of some comorbidities. 2) The onset of symptoms may have occurred before the recorded date, since many of these patients, with cognitive impairment, lack the capacity to express themselves and the symptoms may have gone unnoticed. 3) The non-randomized design of the study may have favored a bias in the selection of patients who received CP, but we believe the controls are reasonably well chosen, as they were taken from a period when CP could not be obtained due to a breach in stock. 4) Patients in the treatment group received remdesivir more often than controls, and this could have improved their outcome. Remdesivir was only administered within 7 days of symptom onset, and in both groups the time from onset of symptoms to hospital admittance was similar (Control 5.2 days vs. Plasma 5.3 days; *p* = 0.877). Thus, we can only speculate that physicians considered that these patients were in worse condition and decided to use both CP and remdesivir. 5) Our patients were treated at dedicated COVID units, but lacked a specific geriatric assessment, that could have helped to reach more informed conclusions.

## Conclusion

CP added to standard therapy in patients over 80 years of age did not significantly reduce overall in-hospital mortality, ICU admission or time to hospital discharge, compared with standard treatment alone. Interpretation is limited by the small sample size and the high percentage of plasma units with low-titer antibodies.

## Data Availability

The datasets used and analyzed during the current study are available from the corresponding author on reasonable request.

## Abbreviations

CP: Convalescent plasma
ICU: Intensive care unit
LoS: Length of stay
LTCF: Long term care facilities
FDA: US Food and Drug Administration
TRALI: Transfusion-associated acute lung injury
ELISA: Enzyme linked immunosorbent assay
IHM: In-hospital mortality
